# Health-Related Behavior and Psychosocial Characteristics of Adolescent Female Smokers in Korea, Compared with Adolescent Male Smokers

**DOI:** 10.3390/healthcare11121707

**Published:** 2023-06-11

**Authors:** Yong-Sook Eo, Yeon-Hee Lee, Myo-Sung Kim

**Affiliations:** 1 College of Nursing, Dongguk University-WISE, Gyeongju 38066, Republic of Korea; nursingeo@dongguk.ac.kr; 2 Department of Nursing, Dong-Eui University, Busan 47340, Republic of Korea; vandi@deu.ac.kr

**Keywords:** adolescents, female, health behavior, psychosocial characteristics, smoking

## Abstract

This study aimed to investigate the relationship between health-related behavior and psychosocial characteristics among adolescent female smokers in South Korea using data from the 17th Korea Youth Risk Behavior Web-based Survey (KYRBWS) conducted in 2021. The analysis participants comprised 2407 adolescent smokers who were currently smoking, out of a sample of 54,835 participants. The characteristics of adolescent female smokers were examined by comparing them with those of adolescent male smokers. The results showed that male and female adolescent smokers accounted for 69.2% and 30.8% of the sample, respectively. Multiple logistic regression analysis identified school type, subjective socioeconomic status, physical activity, breakfast consumption, alcohol consumption, sexual experience, stress, generalized anxiety, and suicidal ideation as significant factors associated with adolescent female smokers. These findings provide important foundational data for the development of smoking-cessation programs and policies tailored specifically to adolescent female smokers.

## 1. Introduction

Smoking is a leading cause of preventable deaths and diseases worldwide [1]. According to the World Health Organization (WHO), smoking causes approximately 8 million deaths annually, including 1.2 million deaths from secondhand smoke exposure, as of 2022 [2]. In Korea, direct smoking caused approximately 58,036 deaths in 2019 (50,942 males and 7094 females), resulting in a socioeconomic cost of 12.2 trillion KRW, which caused increased economic losses and healthcare expenses [3]. Since the implementation of the National Health Promotion Act in 1995, the Korean government has taken institutional measures to reduce smoking rates, such as raising cigarette prices, mandating smoking education, establishing smoke-free areas, and providing various programs through the National Smoking Cessation Support Center [4]. Consequently, the smoking rate among males had declined from 42.2% in 2013 to 31.3% in 2021. However, the smoking rate among females has remained nearly unchanged, at 6.2% in 2013 and 6.9% in 2021 [5].

The smoking rate among adolescents is 4.5%, with males and females at 6.0% and 2.9%, respectively, indicating that the smoking rate among females is approximately half of that among males [6]. Although this rate has decreased from 11.2% in 2019, prior to the COVID-19 pandemic, the male-to-female ratio of smoking adolescents has increased, at 9.3% and 3.8% for males and females, respectively [7]. Particularly concerning is the increasing trend of smoking among female students; however, male students have also shown similar smoking rates in 2021 and 2020, necessitating measures to address this issue. In the East Asian cultural context, female adolescent smokers tend to smoke alone in personal spaces more often than male adolescents do [8], and there is a tendency to conceal or underreport smoking behavior [9,10]. Therefore, smoking among female adolescents is underreported and initiated at an average age of 13.5 years during early adolescence [11], which is a significant health concern.

Smoking during adolescence has a more pronounced impact on cellular and tissue damage, compared with that during adulthood, because of the developmental characteristics of growing adolescents [12]. Exposure to toxic substances in cigarettes, such as nicotine, tar, carbon monoxide, and other carcinogens, can lead to the development of chronic diseases [12], increasing the risk of respiratory problems and cardiovascular diseases, such as hypertension, myocardial infarction, and stroke [13]. One-third of adult smokers who began smoking during adolescence have a higher risk of smoking-related diseases, including lung cancer and cardiovascular diseases, leading to increased mortality [14]. On examining the gender-specific risks of smoking, evidence shows that women face a higher risk of mortality due to lung cancer, oral cancer, and cardiovascular diseases caused by smoking [14,15]. Furthermore, smoking among female adolescents has been found to influence women-specific health issues such as premenstrual syndrome [16] and decreased bone density [17], emphasizing the need for special attention to this population.

Adolescent smoking also affects their mental health. The concentration of cotinine, a nicotine metabolite, has been associated with symptoms of major depressive disorder, generalized anxiety disorder (GAD) [18], stress, suicide attempts, and other mental health problems [19]. Adolescence, characterized by rapid physical development and excessive social demands, is a vulnerable stage in which individuals are more susceptible to temptations such as smoking [20]. Although the psychological characteristics of adolescents may predispose them to smoking [20], the harmful substances associated with smoking can contribute to a vicious cycle of negative effects on mental health [18], which is believed to be more detrimental during adulthood.

Previous research has extensively investigated various smoking-related characteristics among adolescents, including smoking experience and frequency, exposure to secondhand smoke [21], dual-smoking behavior [4,22], smoking-affecting factors [23], and smoking cessation [13]. Additionally, factors associated with smoking, such as health-related [24] and psychosocial characteristics [25,26], were examined with respect to gender. These studies have revealed gender differences in health behaviors such as smoking, alcohol consumption, and physical activity [25], as well as variations in smoking patterns and influential factors based on gender [10]. However, limited research has focused on the psychosocial characteristics of adolescent smokers according to gender. Considering that psychosocial characteristics among adolescents can serve as causal factors for smoking, it is essential to explore this relationship comprehensively.

Therefore, this study aimed to examine the characteristics of adolescent female smokers in South Korea using the raw data from the 17th Korea Youth Risk Behavior Web-based Survey (KYRBWS) conducted in 2021. Using adolescent male smokers as a reference group, this study sought to analyze the health behaviors and psychosocial factors of adolescent female smokers. The specific objectives of this study were as follows:

1. To assess the health-related behaviors and psychosocial characteristics of adolescent female smokers in South Korea.
2. To analyze the factors associated with the health-related behaviors and psychosocial characteristics of adolescent female smokers, using male smoking adolescents as a reference group.

## 2. Methods

### 2.1. Study Design

This secondary data analysis utilized data from the 17th KYRBWS in 2021, in order to compare gender-related factors based on the health-related characteristics and psychosocial characteristics of adolescent smokers [6]. Among the entire dataset, the analysis focused on adolescent smokers who are currently smoking, and the characteristics of female adolescent smokers were analyzed based on male adolescent smokers as a reference.

### 2.2. Data and Study Population

The KYRBWS is an annual survey conducted by the Korea Centers for Disease Control and Prevention since 2005 to assess health behaviors and trends among Korean adolescents. The survey is an anonymous, self-administered online questionnaire, with participation from students in grades 1 to 3 of middle school and those in high school. The survey was conducted in April 2021, and the target population included students enrolled in middle and high schools nationwide. The sampling process involved population stratification, sample distribution, and sample extraction.

In the stratification phase, the national population was divided into 117 strata based on 39 regional districts and school levels (middle schools, general high schools, and specialized high schools). The 39 regional districts were classified as metropolitan cities, medium-sized cities, and rural areas within 17 cities and provinces, considering factors such as geographic accessibility, number of schools and population, living environment, smoking rate, alcohol consumption rate, and other relevant factors.

In the sample distribution phase, 400 middle schools and 400 high schools were selected, and the sampled schools were allocated proportionally to match the composition ratio of the population based on the stratification variables. Sample extraction followed a two-stage cluster sampling method, in which schools were sampled as primary units, and one class per grade was randomly selected from the sampled schools. The survey included 796 participating schools (399 middle schools and 397 high schools). Among these schools, a sample class was selected, and all students in the selected sample comprised a total of 50,066 participants. However, in the final analysis, 54,848 students participated in the survey, resulting in a participation rate of 92.9%. Students who were chronically absent, those with disabilities preventing their participation, and those with dyslexia were excluded [6]. From the overall collected sample, the criteria for inclusion in this analysis were students who responded “yes” to the question “Have you smoked cigarettes on one or more days in the past 30 days?” These students were considered current smokers. The criteria for exclusion were students who had not smoked cigarettes in the past 30 days. Consequently, a total of 2407 participants were included in the analysis, consisting of 1630 male adolescents and 777 female adolescents.

### 2.3. Measures

The 2021 KYRBWS comprised 113 items distributed across 16 domains. Among these items, 19 were selected for analysis: seven items related to demographic characteristics, four assessing smoking-related characteristics, five exploring health-related behaviors, and three examining psychosocial characteristics. In instances where the original dataset contained a large number of categories with uneven distributions, posing challenges in terms of comparability with previous studies, we mitigated this limitation by referring to relevant research conducted on a similar cohort [22] and reconfigured the categories into two to three meaningful groupings.

#### 2.3.1. Demographic and Sociological Characteristics

Demographic and sociological characteristics included gender, school type, academic performance, subjective economic status, change in economic status after COVID-19, residential area, and living arrangements with parents.

Gender was categorized as “male” and “female”. The school type was divided into three categories: middle school, high school, and special high school. Academic performance and subjective economic status were reclassified into three categories: “high” for the highest category, “medium” for the middle categories, and “low” for the lowest categories. Economic status changes due to COVID-19 were categorized as “affected” (significantly worsened due to COVID-19) and “not affected” (no significant change or improvement). Residence area was categorized as “large cities” (metropolitan cities with a population of more than 500,000), “medium and small-sized cities” (total population of less than 500,000 or around 100,000), and “province cities” with a rural area. Living arrangements with parents were categorized as “living with family” and “not living with family”, including cases of living with relatives, boarding, living alone, living in dormitories, and living in childcare facilities.

#### 2.3.2. Smoking-Related Characteristics

The smoking-related characteristics included age at smoking initiation, e-cigarette use, smoking-cessation attempts, and secondhand smoke experience at school. Age at smoking initiation was classified into three categories: “before middle school”, “during middle school”, and “during high school”. E-cigarette use was determined by the question, “In the past 30 days, how many days did you use nicotine-containing e-cigarettes?” Those who responded with “1 day or more” were classified as “yes”. Smoking-cessation attempts were assessed using the question, “Have you attempted to quit smoking in the past 12 months?” Secondhand smoking experience at school was determined by the question “In the past 7 days, how many days did you inhale tobacco smoke from other people smoking indoors at school?” Those who responded with “1 day or more per week” were classified as “yes”.

#### 2.3.3. Health-Related Behavior

Health-related behavioral characteristics included subjective health status, physical activity, breakfast consumption frequency, current alcohol consumption, and sexual experiences. For subjective health status, participants were asked, “How do you rate your health?” Responses such as “very healthy”, “healthy”, and “normally healthy” were classified as “healthy”, while “unhealthy” and “very unhealthy” were classified as “unhealthy”. Physical activity was assessed using the question, “In the last 7 days, how many days did you spend more than 60 min a day on physical activities that increased your heart rate or made you go out of breath?” Responses were categorized as “none in the past 7 days”, “1–4 days per week”, and “5 or more days per week”. Breakfast consumption frequency was determined by the question, “How many days in the past 7 days did you eat breakfast?” Responses were categorized as “did not eat in the past 7 days”, “1–4 days per week”, and “5 or more days per week”. Current alcohol consumption was assessed using the question, “How many days in the past 30 days did you drink one or more drinks of alcohol?” Those who responded with “once or more per month” were classified as “yes”. Sexual experience was determined by the question, “Have you ever had sexual intercourse?” Responses such as “yes” and “no” were classified accordingly.

#### 2.3.4. Psychosocial Characteristics

Psychosocial characteristics examined in this study encompassed GAD, stress, and suicidal ideation. GAD was evaluated using the seven-item generalized anxiety disorder (GAD-7) screening tool. The GAD-7 is a self-report questionnaire specifically designed to identify individuals with GAD and measure the severity of their symptoms. Comprising seven items related to anxiety or worry, respondents rated each item on a scale ranging from 0 to 3. The total GAD-7 score ranges from 0 to 21, with scores falling within different ranges, indicating varying levels of anxiety severity: 0–4 (normal), 5–9 (mild), 10–14 (moderate), and 15–21 (severe) [27]. A score of 10 or higher is considered indicative of GAD, with a reported sensitivity of 89% and a specificity of 82%. Previous studies have demonstrated high internal consistency (Cronbach’s α) for the GAD-7, ranging from 0.915 to 0.924 [28,29]. In this study, the GAD-7 exhibited excellent internal consistency, with a Cronbach’s α value of 0.93.

The perceived level of stress was assessed through a single question: “How much stress do you usually feel?” Participants were given response options ranging from “not at all” to “a little”, which were classified as “no”, while “a lot” and “very much” were classified as “yes”. The stress perception item, consisting of a single question, has been utilized in previous studies as a tool to examine the factors influencing adolescent smoking [30].

Suicidal ideation was assessed using the question, “Have you seriously thought about committing suicide in the past 12 months?” Participants’ responses were categorized either as “no” or “yes” based on their answer to this question.

### 2.4. Data Collection

The Youth Health Behavior Survey (KYRBWS) is an annual publication of data that has been conducted since 2005 to assess the health behaviors and trends among Korean adolescents. During data collection, unique numbers were used to ensure the anonymity and confidentiality of participants’ personal information. Consent to participate in the study was obtained from all participants. This study utilized the 17th KYRBWS statistical data from 2021, which were downloaded and used in accordance with the regulations for the public release and management of raw data.

The 17th KYRBWS survey was conducted through an anonymous self-administered online questionnaire among middle and high school students nationwide as of April 2021. The sampling process was conducted in three stages: population stratification, sample distribution, and sample extraction, as described in the research subjects section.

Prior to data collection, sample schools and sample classes were selected, and student demographics were registered. Survey support teachers from the sample schools were then selected and trained. The survey support teachers explained the importance of the study and the participation process to the participants and conducted the survey with students who submitted signed consent forms. The survey was conducted using an online survey program (accessed through the “Nuri House”), and to facilitate the survey, it was administered using school computers or mobile devices (such as tablet PCs or smartphones). The entire survey process took approximately 45 to 50 min.

### 2.5. Statistical Analysis

This study utilized the raw data from the 17th Youth Health Behavior Survey (KYRBWS), which employed a complex sampling design. To ensure accurate statistical analysis, it is recommended to use statistics that incorporate the complex sampling design. Data analysis was conducted using complex sampling with strata, cluster weights, and finite population correction factors (FPC) to ensure population representativeness. The raw data from the 17th KYRBWS provided integrated strata, cluster variables, weights, and FPC, which were considered. The IBM SPSS Statistics 27.0 program (IBM Corp., Armonk, NY, USA) was used for the analysis, with a significance level of *p* < 0.05 applied for hypothesis testing.

Before conducting the analysis, the basic assumptions for logistic regression analysis, which was used to achieve the objectives of this study, were checked. Logistic regression analysis is a non-linear regression analysis where the independent variables are continuous and the dependent variable has a binary outcome. Unlike discriminant analysis, logistic regression does not require the normality assumption of the sample for the dependent variable with a binary outcome [31]. Discrete independent variables were analyzed by applying dummy coding. To assess the adequacy of the logistic regression model in the sample, the −2 Log Likelihood (2642.594), Chi-square (χ^2^ =385.259, *p* < 0.001), and Hosmer–Lemeshow test (χ^2^ =11.072, *p* = 0.198) were examined, indicating that the model was a good fit.

The specific analysis methods are as follows:

1. The demographic characteristics, smoking-related characteristics, health-related behaviors, and psychosocial characteristics of the participants were analyzed using frequency (sample size) and percentages (applying weights).
2. The demographic characteristics, smoking-related characteristics, health-related behaviors, and psychosocial characteristics of smoking adolescents were analyzed based on gender using the Rao-Scott χ^2^-test.
3. Multiple logistic regression analysis was conducted to analyze health-related behaviors and psychosocial characteristics of adolescent female smokers, with adolescent male smokers as the reference group.

## 3. Results

### 3.1. Participant Demographics and Smoking-Related Characteristics

Table 1 presents the participant demographics and smoking-related characteristics. The distribution of participants across different school levels was as follows: middle school (21.1%), high school (51.6%), and specialized high school (27.3%). The majority of the participants (54.3%) had low academic performance, and most reported their subjective economic status as moderate (68.6%). Regarding the economic impact of COVID-19, 60.9% reported no change, while 39.1% perceived a worsening of their economic situation. The participants primarily resided in small and rural areas (55.4%), and 92.5% lived with their parents.

Regarding smoking-related characteristics, the most common age of smoking initiation was that during middle school (69.7%), and 67.9% of the participants reported using liquid electronic cigarettes. Many adolescents used both liquid electronic and conventional cigarettes. Smoking-cessation attempts were reported by 67.9% of the participants, and 20.5% had experienced secondhand smoke at school.

### 3.2. Participants’ Health-Related Behaviors and Psychosocial Characteristics

Table 2 shows the health-related behaviors and psychosocial characteristics of the participants. Most smoking adolescents (87.7%) perceived themselves as healthy. Regarding physical activity, 50.3% reported engaging in physical activity 1–4 days a week. Breakfast consumption frequency was low, with 69.6% reporting eating breakfast less than 4 days a week. Of the participants, 64.6% reported current alcohol consumption, and 38.7% reported having sexual experiences.

Among the psychosocial characteristics of smoking adolescents, the average GAD score was 5.25 ± 5.44. Most participants (39.9%) fell within the normal range, but 25.0% had moderate or higher levels of anxiety. A significant number of participants (50.3%) reported experiencing stress, and 25.0% reported having suicidal ideation.

### 3.3. Gender Differences in Characteristics of Smoking Adolescents

Table 3 illustrates the gender differences in demographic and smoking-related characteristics among smoking adolescents. Significant differences were observed in school type (χ^2^ = 48.588, *p* < 0.001), subjective economic status (χ^2^ = 22.924, *p* < 0.001), changes in economic status due to COVID-19 (χ^2^ = 14.192, *p* < 0.001), and co-residence with parents (χ^2^ = 5.378, *p* = 0.039). However, no significant differences were found in the smoking-related characteristics.

Gender differences in health-related behavioral characteristics showed significant differences in subjective health status (χ^2^ = 20.492, *p* < 0.001), physical activity (χ^2^ = 135.747, *p* < 0.001), breakfast consumption frequency (χ^2^ = 33.848, *p* < 0.001), current alcohol consumption (χ^2^ = 9.002, *p* = 0.002), and sexual experience (χ^2^ = 21.964, *p* < 0.001). Psychosocial characteristics also exhibited differences in GAD (χ^2^ = 61.511, *p* < 0.001), perceived stress (χ^2^ = 105.218, *p* < 0.001), and suicidal ideation (χ^2^ = 125.057, *p* < 0.001) (Table 4).

### 3.4. Smoking-Related Factors among Adolescent Female Smokers

To examine the characteristics of adolescent female smokers among Korean smoking adolescents, a multiple logistic regression analysis was conducted, with adolescent male smokers serving as the reference group (Table 5). The analysis designated demographic, health-related characteristics, and psychosocial characteristics as independent variables and smoking characteristics based on gender as the dependent variable, examining statistically significant results through cross-analysis.

The analysis revealed statistically significant variables for demographic characteristics, health-related behaviors, and psychosocial characteristics. Among the demographic characteristics, compared with adolescent male smokers, females in high school had a lower odds ratio (OR) of 0.67 (95% CI = 0.43–1.04), and females in middle school had a higher odds ratio of 1.54 (95% CI = 0.96–2.48). Regarding subjective economic status, rather than being in the “high” group, females were more likely to be in the “middle” group with an odds ratio of 2.50 (95% CI = 1.69–3.69) and in the “low” group with an odds ratio of 2.00 (95% CI = 1.27–3.13).

Regarding health-related behaviors, females had lower odds ratios in physical activity compared with the “no activity” group: 0.43 (95% CI = 0.34–0.54) for the “1–4 days per week” group and 0.19 (95% CI = 0.13–0.28) for the “5 or more days per week” group. Breakfast consumption frequency showed that females had lower odds ratios compared with the “no consumption” group: 0.96 (95% CI = 0.76–1.21) for the “1–4 days per week” group and 0.67 (95% CI = 0.47–0.86) for the “5 or more days per week” group. Current alcohol consumption and sexual experience were both higher among females compared with males, with odds ratios of 1.34 (95% CI = 1.07–1.68) and 1.34 (95% CI = 1.05–1.71), respectively.

Among the psychosocial characteristics, females had higher odds ratios for GAD compared with the “normal” group: 1.16 (95% CI = 0.89–1.50) for the “mild” group, 1.52 (95% CI = 1.06–2.18) for the “moderate” group, and 1.93 (95% CI = 1.27–2.92) for the “severe” group. Perceived stress was lower among females in the “yes” group than in those in the “no” group, with an odds ratio of 0.74 (95% CI = 0.58–0.94). Females had higher odds ratios for suicidal ideation compared with the “no” group, with an odds ratio of 1.69 (95% CI=1.34–2.13).

## 4. Discussion

This study examined the health-related behaviors and psychosocial characteristics of female adolescent smokers using data from the 17th KYRBWS (2021). Among the 2407 adolescent smokers, 69.2% were male and 30.8% were female. No significant differences in smoking-related characteristics were found based on the gender of smoking adolescents. Comparing our findings with those of a study by Lee and Song [13], which explored factors related to smoking-cessation attempts by gender among smoking adolescents using data from the 16th Youth Health Behavior Survey (2020), similar results were observed. However, variations between genders were observed regarding the age of smoking initiation, e-cigarette use, and exposure to indirect smoking at school.

In 2020, the proportion of smoking initiation before middle school was 15.7% (17.7% for boys and 10.6% for girls) [13]. By 2021, this proportion increased to 16.6%, with a decrease of 17.1% for boys and an increase to 15.4% for girls. These findings suggest that girls tend to initiate smoking at a relatively younger age compared to boys. Furthermore, the rate of e-cigarette use significantly increased from 41.8% in 2020 (35.7% for boys and 40.0% for girls) [13] to 79.4% in 2021 (79.8% for boys and 78.5% for girls). Smoking-cessation attempts decreased from 69.6% in 2020 (69.2% for boys and 69.4% for girls) [13] to 67.9% in 2021 (68.3% for boys and 66.8% for girls), with a greater decrease observed among girls.

These findings align with those by Kim [32], who reported lower smoking-cessation attempt rates and higher failure rates among women [33,34]. Kim [32] attributed the higher failure rates among women to concerns regarding menstrual cycles, weight gain, and depression. Additionally, lack of family support due to the stress caused by withdrawal symptoms during smoking cessation, which often went unnoticed by family members, also played a role. A review [15] indicated that women face more challenges in achieving long-term smoking cessation compared with men. The interpretation of this gender difference in epidemiological studies varies, considering multiple biological, psychological, and social factors across different times and locations. Therefore, gaining a comprehensive understanding of the smoking behaviors of female adolescent smokers is crucial for the development of effective intervention strategies targeting this population.

Regarding school level, high school students (78.9%) were found to smoke more than middle school students (21.1%), which is consistent with previous studies [30,35]. However, the proportion of middle school girls among female adolescent smokers was significantly higher (28.9%) than that of boys (17.7%). According to the 2021 Youth Smoking Rate Trends Survey conducted by the Korea Centers for Disease Control and Prevention, the overall smoking rates among male and female students were 6.0% and 2.9%, respectively. Among high school students, the smoking rate was 10.0% among boys, which was more than twice the rate among girls (4.2%). However, among middle school students, the difference was smaller, with smoking rates of 2.1% and 1.6% among boys and girls, respectively. These findings support that the proportion of male adolescent smokers is higher than that of female adolescent smokers; however, the gender difference gradually decreased in middle schools.

A study analyzing smoking-cessation trends among 50,228 smokers from 17 European countries found that those who started smoking before the age of 16 were less likely to quit smoking [36]. Another study of 2536 Malaysian adolescents revealed that those who started using e-cigarettes before the age of 14 had a 1.3 times lower smoking-cessation attempt rate than those who started using e-cigarettes after the age of 14 [37]. Furthermore, younger age at smoking initiation is associated with lower quitting rates [38] and a higher risk of relapse after quitting [39]. Therefore, additional efforts should be made to prevent female adolescent smokers from starting to smoke, and tobacco control strategies should be implemented to delay or deter smoking initiation in this population [36].

Compared with male adolescent smokers, female adolescent smokers had an intermediate or low subjective socioeconomic status, and a higher proportion of female students experienced economic difficulties due to COVID-19. However, in the multivariate analysis, subjective economic status emerged as the only significant variable, indicating that the perception of household economic level during normal times is more important than the short-term changes in economic status due to crises such as COVID-19. Previous studies have identified both low [40,41] and high [37] household socioeconomic status as factors influencing adolescent smoking, with many consistently reporting that a higher allowance [35,41] is associated with lifetime and current smoking. Therefore, it is necessary to avoid categorizing adolescents from low socioeconomic backgrounds as being at risk of smoking.

Green et al. [40] reported that smoking typically begins during adolescence and is patterned based on socioeconomic position. They found that socioeconomic disadvantages were associated with higher rates of smoking initiation and escalation among British adolescents aged 11–15, with greater inequality in smoking initiation observed at younger ages, suggesting that interventions should focus on reducing inequality in smoking initiation among early adolescents. Gwon and Jeong [41] reported that adolescents who received more allowances were more likely to engage in lifetime and current smoking. They found that a higher household economic status was associated with a higher likelihood of lifetime smoking, and current smoking was more likely among those from households with varying or higher economic statuses. As a certain level of economic resources is required for adolescents to purchase tobacco products, it has been reported that adolescents who smoke either receive a larger allowance from their parents or purchase cigarettes through means such as part-time jobs. Therefore, prevention and smoking-cessation education for female adolescent smokers should strategically consider various factors, including socioeconomic status and participation in part-time jobs.

Regarding health-related behaviors, a lower proportion of female adolescent smokers engaged in physical activity and ate breakfast, compared with male adolescent smokers. This finding is similar to that of Kaczynski et al. [42], who reported that male adolescent non-smokers engage in more physical activity to reduce the harmful effects of smoking and that skipping breakfast is associated with smoking, alcohol use, and sedentary behavior. The results of this study support those of previous research indicating gender differences in breakfast-skipping [43]. A study conducted on students (*n* = 318) in grades 9 through 12 in three schools in southwestern Ontario found a significant difference between male non-smokers (60.4%) and currently smoking students (31.9%) in terms of eating breakfast daily; however, there was no statistical difference among female students, who had concerns about weight gain [43].

Korean adolescent smokers were found to have a significantly lower likelihood of consuming fruits, vegetables, and dairy products; however, they consumed significantly more fast food compared with non-smokers [44]. Adolescence is an important period for the formation of health-related habits such as exercise and proper nutrition, which have long-term effects on lifelong health [45]. Adolescents may lack deep awareness of health-related issues, leading to neglect of health management and engagement in unhealthy behaviors. This study found that female adolescent smokers had lower rates of health-promoting behaviors such as physical activity and eating breakfast compared with male adolescent smokers. Therefore, paying attention to female adolescent smokers and supporting them in developing healthy lifestyles is important. In particular, nutritional deficiencies related to reproductive health promotion is necessary in addition to the increased nutritional requirements during adolescence.

Smoking among adolescents not only poses health risks but is also correlated with other addictive behaviors and deviant actions, such as alcohol consumption and drug abuse [30,46,47]. The association between smoking and alcohol consumption is well documented, with higher alcohol consumption being positively linked to increased smoking rates [48]. A study investigating the risk ratio of smoking in relation to health behaviors revealed that individuals with a history of alcohol consumption had a 7.5 times higher risk of smoking. Furthermore, as alcohol consumption frequency increased, the smoking rate increased, reaching 82.6% among adolescents who reported almost daily alcohol consumption [35]. Research that examines the connection between smoking and sexual behavior indicates that smoking is associated with a 1.47 times higher likelihood of early sexual intercourse among Ethiopian women aged 15–24 [49]. In Korea, female adolescent smokers were found to exhibit behavioral characteristics linked to the use of oral contraceptives [50], emphasizing the necessity of approaching the challenges faced by female adolescent smokers from a reproductive health perspective. Particularly in Korean society, where female smoking is often viewed negatively, female adolescent smokers may conceal their smoking behavior [9]. Therefore, this study shows that female adolescent smokers exhibited higher rates of alcohol consumption and sexual experiences compared with their male counterparts, which underscores the importance of thoroughly examining not only their health-risk behaviors, but also their deviant actions.

Female adolescent smokers showed lower stress levels but higher rates of GAD and suicidal ideation, compared with male adolescent smokers. Previous studies have found that psychological and social characteristics such as stress [13,25,51,52], anxiety [25,52], and suicide [51] were statistically related to adolescent smoking behavior. Wiggert et al. [53] reported that anxiety plays an important role in smoking tendencies and relapse, and that smoking cravings increase in response to stress among individuals with high trait anxiety. Research shows that adolescents smoke to regulate unpleasant emotions, such as stress, anxiety, and suicidal thoughts, and to obtain temporary emotional stability [35]. Particularly in Korea, recent social factors such as educational culture focused on entrance exams, weakened family bonds, and unguarded exposure to addictive media have exacerbated psychological and social problems of adolescents [51]. While female adolescent smokers choose smoking to gain temporary emotional stability and alleviate their psychological and social problems, their adolescent characteristics of viewing smoking as a rebellious behavior can worsen anxiety and stress reactions. Specifically, the higher prevalence of GAD among female adolescent smokers in this study indicates the need to recognize it as a psychological problem characterized by difficulty in control, persistent worry, and anxiety [54], rather than perceiving them as passive personalities or well-behaved students, which makes early detection difficult [55]. There is an urgent need to understand the psychological and social characteristics of female adolescent smokers and provide the necessary interventions, especially considering their high suicidal ideation.

This study offers valuable insights into the relationship between health-related behaviors and the psychological and social characteristics specific to female adolescent smokers, emphasizing the significance of incorporating sex-specific factors in evaluating such aspects. Based on these findings, it is evident that the development and implementation of smoking-cessation programs for adolescent smokers should address sex-specific smoking-related factors.

## 5. Limitations

It is important to note that this study was based on cross-sectional data from a single year, limiting its ability to establish causality, despite identifying associations between variables. Moreover, the use of secondary data variables has certain limitations. Therefore, future research should aim to conduct longitudinal studies that explore the causal factors influencing smoking among female adolescent smokers, considering diverse variables such as parental smoking, peer smoking, depression, and other factors related to adolescent smoking.

Furthermore, the stress variable used as a psychosocial characteristic factor in this study consisted of a single question, which may compromise the validity of the tool. Although it has been used as a factor influencing adolescent smoking in previous secondary data analyses [30], it is necessary to confirm the relationship with female adolescent smokers using a tool with higher validity and reliability in the future.

## 6. Conclusions

This study examined the smoking-related characteristics of female adolescent smokers compared with those of their male counterparts. The findings revealed that the majority of female adolescent smokers were middle school students, with a significant portion reporting subjective socioeconomic statuses categorized as intermediate or low. They exhibited lower levels of physical activity and breakfast consumption, while demonstrating higher rates of health-risk behaviors such as current alcohol consumption and sexual activity. Moreover, female adolescent smokers showed a higher prevalence of GAD compared with male adolescent smokers, and the smoking rate increased with higher levels of anxiety. Although they perceived lower stress levels, they also reported a higher frequency of suicidal ideation. Consequently, comprehensive health education programs should be designed to encompass reproductive health promotion behaviors, including physical activity, breakfast habits, alcohol consumption, and sexual education. Moreover, attention should be paid when addressing psychological and social concerns, such as anxiety and suicide. To improve smoking prevention among female adolescent smokers, it is necessary to develop tobacco control strategies and programs to delay or deter smoking initiation in this population.

## Figures and Tables

**Table 1:** Demographic and smoking-related characteristics of the participants (*n* = 2407).

Variables	Categories	*n* (%)
School	Middle school	570 (21.1)
	High school	1192 (51.6)
	Specialized high school	645 (27.3)
Academic performance	High	194 (8.0)
	Middle	904 (37.7)
	Low	1309 (54.3)
Perceived SE status	High	274 (11.9)
	Middle	1663 (68.6)
	Low	470 (19.5)
COVID-19 related SE change	Yes	952 (39.1)
	No	1455 (60.9)
Region of residence	Metropolitan city	940 (38.2)
	Medium and small city	1254 (55.4)
	Province	213 (6.4)
Residential with parents	None	189 (7.5)
	Yes	2218 (92.5)
Time of initiation smoking	Before middle school	381 (16.6)
(*n* = 2400)	Middle school	1681 (69.7)
	High school	338 (13.7)
Use electronic liquid cigarette	None	515 (20.6)
	Yes	1892 (79.4)
Attempt smoking cessation	None	763 (32.1)
(*n* = 2403)	Yes	1640 (67.9)
Second-hand smoking in school	None	1934 (79.5)
	Yes	473 (20.5)

*n* (%) = *n*: unweighted; %: weighted; SE = socioeconomic.

**Table 2:** Health-related behaviors and psychosocial characteristics of the participants (*n* = 2407).

Variables	Categories	M ± SD or *n* (%)
Subjective health status	Healthy	2112 (87.7)
	Unhealthy	295 (12.3)
Physical activity per week	None	758 (32.0)
	1–4 days	1205 (50.3)
	≥5 days	444 (17.7)
Frequency of eating breakfast per week	None	691 (27.9)
	1–4 days	1005 (41.7)
	≥5 days	711 (30.4)
Current alcohol consumption	None	855 (35.4)
	Yes	1552 (64.6)
Sexual activity	None	1489 (61.3)
	Yes	918 (38.7)
GAD (*n* = 1821)	M±SD	5.25 ± 5.44
	Normal	740 (39.9)
	Mild	624 (35.1)
	Moderate	264 (14.3)
	Severe	193 (10.7)
Perceived stress	None	1201 (49.7)
	Yes	1206 (50.3)
Suicidal ideation	None	1804 (75.0)
	Yes	603 (25.0)

*n* (%) = *n*: unweighted; %: weighted; M ± SD = Mean ± Standard deviation; GAD = Generalized Anxiety Disorder.

**Table 3:** Demographic and smoking-related characteristics by gender among smoking adolescents (*n* = 2407).

Variables	Categories	Male (*n* = 1630) *n* (%)	Female (*n* = 777) *n* (%)	Rao-Scott χ^2^ (*p*)
Demographic characteristics				
School type	Middle school	331 (17.7)	239 (28.9)	48.588 (<0.001)
	High school	888 (55.7)	304 (42.5)	
	Specialized high school	411 (26.6)	234 (28.6)	
Academic	High	138 (8.4)	56 (7.2)	0.960 (0.638)
performance	Middle	615 (37.7)	289 (37.7)	
	Low	877 (53.9)	432 (55.1)	
Perceived SE status	High	215 (13.8)	59 (7.6)	22.924 (<0.001)
	Middle	1120 (68.1)	543 (69.6)	
	Low	295 (18.1)	175 (22.8)	
COVID-19 related	Yes	597 (36.6)	355 (44.7)	14.192 (<0.001)
SE change	No	1033 (63.4)	422 (55.3)	
Region of residence	Metropolitan city	648 (39.2)	292 (35.8)	4.018 (0.383)
	Medium and small city	829 (54.1)	425 (58.5)	
	Province	153 (6.7)	60 (5.7)	
Residential with	Yes	1512 (93.4)	706 (90.7)	5.378 (0.039)
Parents	None	118 (6.6)	71 (9.3)	
Smoking-related characteristics				
Time of initiation Smoking (*n* = 2400)	Before middle school	270 (17.1)	111 (15.4)	2.572 (0.306)
	Middle school	1140 (69.8)	541 (69.3)	
	High school	214 (13.1)	124 (15.3)	
Use electronic liquid	None	337 (20.2)	178 (21.5)	0.539 (0.447)
cigarette	Yes	1293 (79.8)	599 (78.5)	
Attempt smoking	None	517 (31.7)	246 (33.2)	0.467 (0.532)
cessation(*n* = 2403)	Yes	1111 (68.3)	529 (66.8)	
Second-hand	None	1312 (79.8)	622 (78.8)	0.303 (0.632)
smoking in school	Yes	318 (20.2)	155 (21.2)	

*n* (%) = *n*: unweighted; %: weighted; SE = socioeconomic.

**Table 4:** Health-related behaviors and psychosocial characteristics by gender among smoking adolescents (*n* = 2407).

Variables	Categories	Male (*n* = 1630) *n* (%)	Female (*n* = 777) *n* (%)	Rao-Scott χ^2^ (*p*)
Health-related behaviors				
Subjective health status	Healthy	1465 (89.7)	647 (83.1)	20.492 (<0.001)
	Unhealthy	165 (10.3)	130 (16.9)	
Physical activity per week	None	403 (25.2)	355 (47.4)	135.747 (<0.001)
	1–4 days	849 (53.1)	356 (43.8)	
	≥5 days	378 (21.7)	66 (8.8)	
Frequency of eating breakfast	None	449 (26.1)	242 (31.9)	33.848 (<0.001)
per week	1–4 days	642 (39.8)	363 (45.8)	
	≥5 days	539 (34.1)	172 (22.3)	
Current alcohol consumption	None	621 (37.3)	234 (31.0)	9.002 (0.002)
	Yes	1009 (62.7)	543 (69.0)	
Sexual activity	None	1061 (64.4)	428 (54.4)	21.964 (<0.001)
	Yes	569 (35.6)	349 (45.6)	
Psychosocial characteristics				
GAD (*n* = 1821)	Normal	520 (45.0)	220 (30.9)	61.511 (<0.001)
	Mild	389 (35.5)	235 (34.5)	
	Moderate	128 (11.7)	136 (18.9)	
	Severe	83 (7.8)	110 (15.7)	
Perceived stress	None	696 (42.8)	505 (65.4)	105.218 (<0.001)
	Yes	934 (57.2)	272 (34.6)	
Suicidal ideation	None	1336 (81.6)	468 (60.2)	125.057 (<0.001)
	Yes	294 (18.4)	309 (39.8)	

*n* (%) = *n*: unweighted; %: weighted; GAD = Generalized Anxiety Disorder.

**Table 5:** Multiple logistic regression for predictors of female adolescents with smoking (*n* = 2407).

Variables	Categories	aOR	(95% CI)	*p*
Demographic characteristics				
School type	Specialized high school (Ref.)	1		<0.001
	High school	0.67	(0.43, 1.04)	
	Middle school	1.54	(0.96, 2.48)	
Perceived SE status	High (Ref.)	1		<0.001
	Middle	2.50	(1.69, 3.69)	
	Low	2.00	(1.27, 3.13)	
COVID-19 related SE change	Yes (Ref.)	1		0.100
	No	0.82	(0.65, 1.04)	
Residential with parents	Yes (Ref.)	1		0.495
	No	0.85	(0.53, 1.36)	
Health-related behaviors				
Subjective health status	Unhealthy (Ref.)	1		0.678
	Healthy	1.07	(0.79, 1.45)	
Physical activity per week	None (Ref.)	1		<0.001
	1–4 days	0.43	(0.34, 0.54)	
	≥5 days	0.19	(0.13, 0.28)	
Frequency of eating breakfast per week	None (Ref.)	1		0.003
	1–4 days	0.96	(0.76, 1.21)	
	≥5 days	0.67	(0.47, 0.86)	
Current alcohol consumption	None (Ref.)	1		0.012
	Yes	1.34	(1.07, 1.68)	
Sexual activity	None (Ref.)	1		0.021
	Yes	1.34	(1.05, 1.71)	
Psychosocial characteristics				
GAD	Normal (Ref.)	1		0.013
	Mild	1.16	(0.89, 1.50)	
	Moderate	1.52	(1.06, 2.18)	
	Severe	1.93	(1.27, 2.92)	
Perceived stress	None (Ref.)	1		0.014
	Yes	0.74	(0.58, 0.94)	
Suicidal ideation	None (Ref.)	1		<0.001
	Yes	1.69	(1.34, 2.13)	

Notes. The reference group was male adolescents with smoking; aOR = adjusted odds ratio, CI = 95% confidence interval; GAD = Generalized Anxiety Disorder; SE = socioeconomic; Ref = reference.

## Data Availability

No new data were created or analyzed in this study. Data sharing is not applicable to this article.
